# CNS disease associated with enhanced type 1 interferon signalling

**DOI:** 10.1016/S1474-4422(24)00263-1

**Published:** 2024-11-01

**Authors:** Yanick J Crow

**Affiliations:** 1 MRC Human Genetics Unit, Institute of Genetics and Cancer, University of Edinburgh, Edinburgh, EH4 2XU, UK; 2 Laboratory of Neurogenetics and Neuroinflammation, Imagine Institute, INSERM UMR1163, 75015, Paris, France

## Abstract

The ability to mount an interferon-mediated innate immune response is essential in protection against neurotropic viruses, but antiviral type 1 interferons also have neurotoxic potential. Production of type 1 interferons can be triggered by self-derived nucleic acids and the brain can be susceptible to inappropriate upregulation of type 1 interferon signalling. Homeostatic regulation of type 1 interferons has been implicated in the context of both rare inborn errors of immunity, referred to as the type 1 interferonopathies, and more common neurodegenerative disorders such as Parkinson’s disease, Alzheimer’s disease and amyotrophic lateral sclerosis. Recent developments include new insights into the pathogenesis of these disorders seen in both paediatric and adult practice, as well as into their diagnosis and management. The role of type 1 interferons in brain cellular health implies the future therapeutic potential of approaches that target these interferons and their signalling.

## Introduction

Type I interferons are fundamental to protect humans from external pathogens (1). Possibly all vertebrate nucleated cells can both make and respond to type 1 interferons (2) and deficits in type 1 interferon signalling are associated with a susceptibility to viral infection (3). Notably, type 1 interferon mediated antiviral signalling appears to be particularly relevant in the CNS, as evidenced by an increased frequency of herpes simplex virus 1 (HSV-1) encephalitis in individuals who have loss-of-function mutations in molecules involved in type 1 interferon induction in response to the presence HSV-1 (4), or of West Nile virus encephalitis in individuals who develop antibodies that neutralise type 1 interferons (5, 6) (these antibodies also explaining an important proportion of adverse reactions to yellow fever virus live-attenuated vaccine (7)). Conversely, enhanced type 1 interferon signalling is potentially deleterious to cellular health, and the brain appears to be particularly vulnerable: type 1 interferons have neurotoxic potential, as shown by the iatrogenic neurological sequelae of exposure to exogenous interferon (8, 9). Further evidence of such neurotoxicity comes from the CNS involvement seen in certain type 1 interferonopathies, a grouping of monogenic disorders where tissue damage is suggested to be directly attributable to inappropriately enhanced type 1 interferon signalling (10). In this Personal View, I aim to provide an overview of these disorders, covering the physiological role of type 1 interferon signalling, the characteristics, diagnosis, and management of type 1 interferonopathies, and the potential role of type 1 interferons in neurodegenerative diseases.

### Type 1 interferon signalling

The 16 human type 1 interferons (12 IFN-α, and one each of IFN-β, IFN-ω, IFN-κ and IFN-ε) all bind the same heterodimeric receptor composed of IFNAR1 and IFNAR2 (respectively, IFN-α and β receptor subunits 1 and 2), resulting in a powerful transcriptomic response involving the expression of hundreds of interferon stimulated genes mediated through the JAK-STAT (Janus kinase-signal transducer and activator of transcription) signalling pathway (11). In turn, the interferon stimulated gene encoded proteins play diverse roles in modifying the innate and adaptive immune systems, as well as cell growth, proliferation and death - and thus pathogen survival (12).

**Figure 1*:**
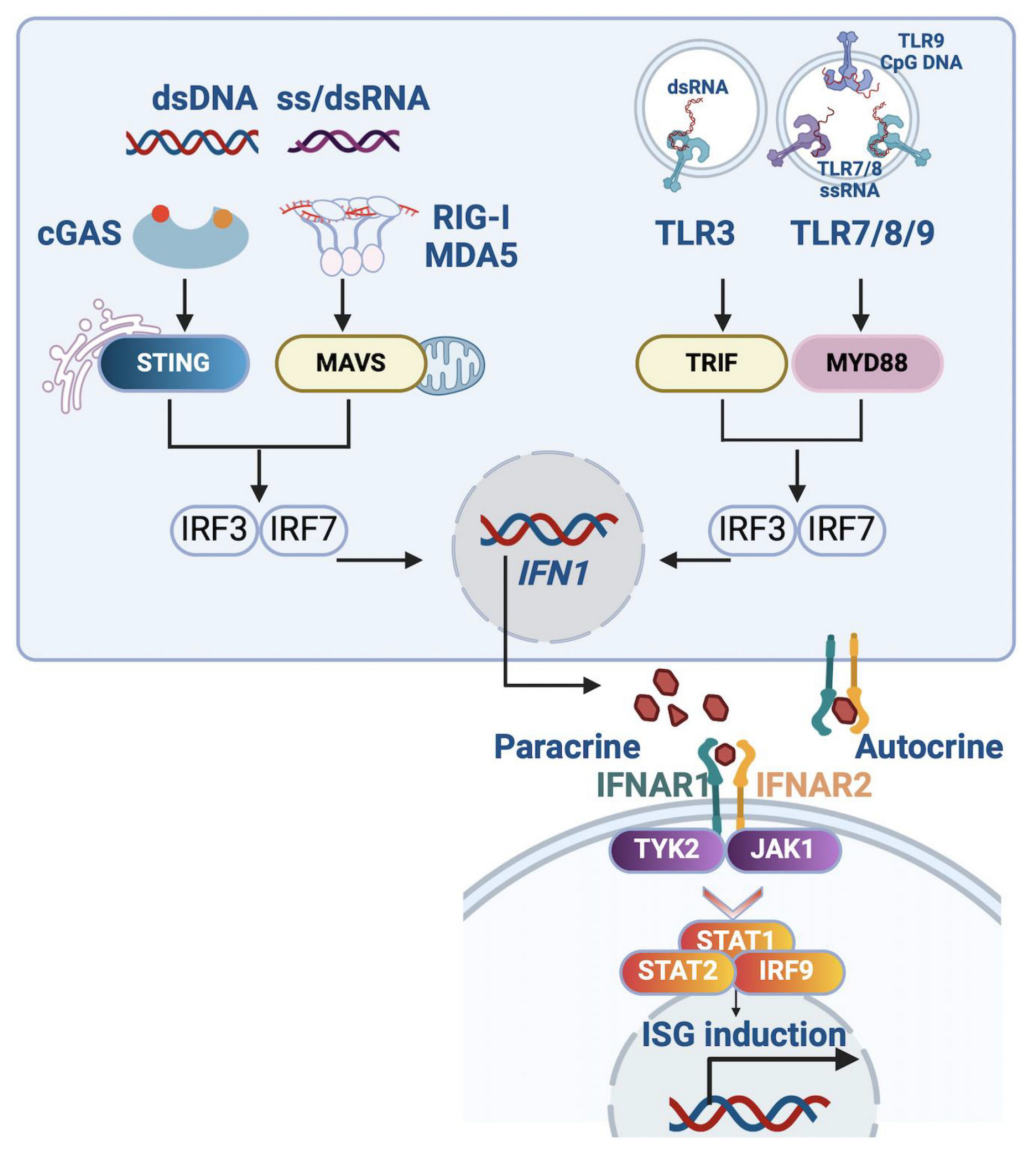
Type I interferon signalling Type I interferon (IFN) signaling elicited by nucleic acids involves cytosolic and endosomal nucleic acid sensors, signal transducers and transcriptional effectors, ultimately leading to the synthesis of type I IFNs. Cytosolic sensors include the ss/dsRNA retinoic acid-inducible gene I (RIG-I)-like receptors (RLRs) RIG-I and melanoma differentiation-associated protein 5 MDA5 (encoded by *IFIH1*), and the DNA sensor cyclic cGAS (GMP–AMP synthase). The Toll-like receptors (TLRs) TLR3, TLR7, TLR8 and TLR9 sense dsRNA (TLR3), ssRNA (TLR7/8) and DNA (TLR9) in endosomes. On secretion, type 1 interferons mediate autocrine and paracrine effects by binding to IFN-α and β receptor subunit 1 (IFNAR1) and 2 (IFNAR2) heterodimers, which initiate a signal transduction cascade culminating in the transcription of interferon stimulated genes (ISGs). CGAS, cyclic GMP-AMP synthase; dsDNA, double-stranded DNA; dsRNA, double-stranded RNA; IFN1, type I IFN; IRF, interferon regulatory factor; JAK1, Janus kinase 1; MAVS, mitochondrial antiviral signaling protein; MDA5, melanoma differentiation associated protein 5; MYD88, myeloid differentiation primary response 88; RIG-I, retinoic acid-inducible gene I; ssRNA, single-stranded RNA; STAT, signal transducer and activator of transcription; STING, stimulator of interferon genes; TLR, Toll-like receptor; TRIF (TICAM), TIR domain-containing adaptor molecule 1; TYK2, tyrosine kinase 2. Note that, while not shown here, engagement of cytosolic and endosomal TLR receptors also triggers NF-kB (nuclear factor kappa B subunit 1) induction, and the subsequent production of inflammatory cytokines. ^*^Created with BioRender.

Remarkable progress has been made in the last 20 years in defining the pathways involved in initiating, transducing, regulating and resolving type 1 interferon signalling. In brief, the mammalian immune response to viral infection involves host-encoded nucleic acid receptors that recognise the presence of viral (‘foreign’) RNA and DNA, both in the cytosol and within the endosomal compartment (Figure 1). Importantly, these same nucleic acid receptors also have the potential to sense self-derived nucleic acids (13–16), thereby inducing antiviral signalling in the absence of virus. While such signalling might have physiological relevance in ensuring a tonic level of protection against virus (see below), or as a response to tumour (17), chronic induction of antiviral signalling results in autoinflammation (18–20). In the context of people who have mutations in the genes that affect type 1 interferon signalling, the resultant (overlapping) clinical phenotypes, referred to as the type 1 interferonopathies, highlight the physiological importance of self versus non-self nucleic acid discrimination.

### Type I interferonopathies

51 discrete mutant genotypes are currently classified as causing type 1 interferonopathies (21), with close to half of these characterised by significant involvement of the CNS (Table 1). Several comprehensive reviews exploring the pathways linking mutant genotype and enhanced type 1 interferon signalling are available (10, 16, 22, 23). In brief, the largest grouping of type I interferonopathies relates to a disturbance of the regulation of nucleic acid homeostasis, whereby abnormal quantity, location, modification or sensing of endogenous RNA and DNA ligands results in inappropriate induction of type 1 interferons. A second important subgroup is accounted for by mutations in genes involved in type 1 interferon signalling per se – for example, further recent descriptions of defects in negative feedback regulation of the type I interferon pathway (24–26). In particular, mitochondria represent a source of immunostimulatory nucleic acids, with a diversity of nucleic acid forms generated during the transcription and replication of mitochondrial DNA. Mutations in PNPT1 (polyribonucleotide nucleotidyltransferase 1) represent the first described Mendelian mitochondrial cytopathy associated with enhanced type 1 interferon signalling (27, 28), showing notable phenotypic overlap with the first described type I interferonopathy Aicardi-Goutières syndrome (AGS) (29, 30). PNPT1 is a component of the mitochondrial degradosome, responsible for the decay of one of the two RNA strands generated during bidirectional transcription of mitochondrial circular RNA. Biallelic loss-of-function mutations in PNPT1 lead to the egress of mitochondrial double-stranded RNA into the cytoplasm, and the subsequent induction of type I interferon and enhanced interferon stimulated gene expression mediated by the cytoplasmic dsRNA receptor MDA5. Pathogenic mutations in ubiquitously expressed ATAD3A (ATPase family AAA domain–containing protein 3A) were shown to cause an upregulation of type I interferon signalling through the dsDNA sensor cGAS, resulting from a leakage of mtDNA into the cytoplasm (31). The observation that damaged mitochondria are prone to release both mitochondrial RNA and DNA, which can induce type 1 interferon signalling, raises the possibility that innate immune system engagement might contribute to the phenotype of other monogenic mitochondrial-related disorders (32). As discussed further below, type 1 interferon induction by mitochondrial-derived nucleic acid has also been implicated in a number of common neurodegenerative diseases.

Most type 1 interferonopathy-associated genotypes have emphasised the importance of cytosolic signalling, but data published since 2022 have demonstrated the potential for aberrant engagement of endosomal Toll-like receptor (TLR) signalling to cause autoinflammatory brain disease. Specifically, Brown et al. reported heterozygous gain-of-function mutations in TLR7 to cause recurrent hemichorea requiring treatment with haloperidol in the context of a systemic lupus-like phenotype, also describing a patient with an exclusively neurological presentation i.e. of neuromyelitis optica with positivity for AQP4 autoantibodies (33) (with a well-recognised causal link between neuromyelitis optica and enhanced type 1 interferon signalling (34)). Furthermore, David et al. observed cerebral vasculitis and cerebral calcification in a female proband, developmental delay, epilepsy and marked intracranial calcification in her brother, and a third patient demonstrating cerebral calcification, atrophy and white matter disease (which was progressive despite an apparently excellent response of life-threatening immune cytopenias to haematopoietic stem cell transplantation) (35). Interestingly, neurological involvement is apparently not typical of gain-of-function mutations in UNC93B1 (Unc-93 Homolog B1 - an intracellular chaperone of endosomal TLRs) associated with enhanced TLR7 signalling, having been described in only one case to date (36).

### Clinical and neuroradiological phenotypes

The most frequent neurological signs in the type I interferonopathies involving the CNS are spasticity and dystonia, presenting either as isolated spasticity or, more often, as a combined spastic-dystonia. While most mutant genotypes are also associated with major developmental delay, intellect can be relatively, or even completely, preserved. Importantly, development can be normal for several months up until the onset of neurological disease. Epilepsy is usually only present in early-onset classic AGS, and is relatively easily controlled. Non-neurological stigmata, particularly chilblain-like vasculitic lesions of the extremities (toes, fingers, ears, nose), and glaucoma, can represent vital clues to the diagnosis of these disorders. A number of recognisable clinical scenarios are outlined in Panels 1 and 2.

**Figure 2:**
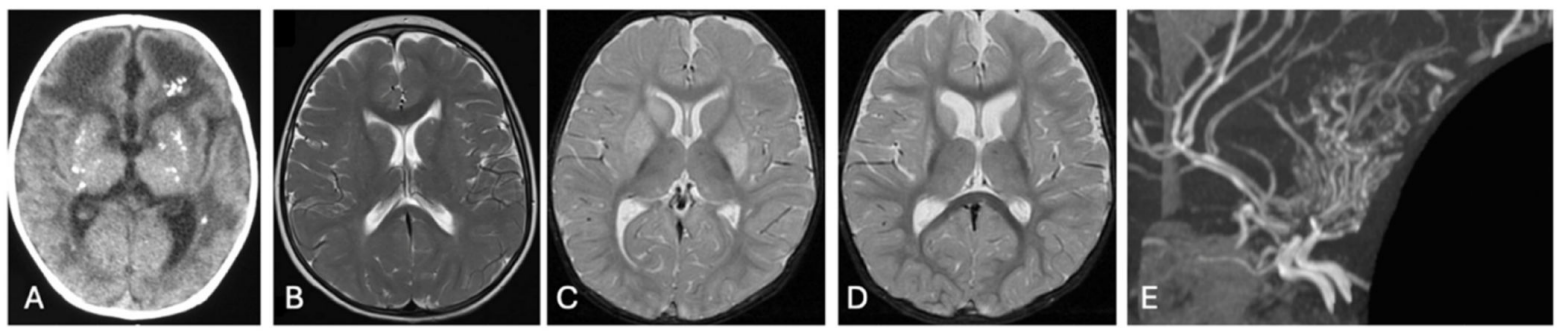
Characteristic imaging features seen in type I interferonopathies A. Axial cranial CT at age 1 year of a child with biallelic loss-of-function mutations in TREX1 showing characteristic calcification of the basal ganglia and extensive white matter abnormalities particularly frontally. B. Cranial axial T2 MRI at age 6 years of a child with a heterozygous gain-of-function mutation in IFIH1 with severe four-limb spasticity but only minimal posterior periventricular white matter high signal abnormalities. C. Cranial axial T2 MRI at age 8 months of a child with biallelic loss-of-function mutations in ADAR1 showing high signal change in the basal ganglia bilaterally. D. Same child as in C at age 2 years 11 months showing marked shrinkage of the basal ganglia. E. MRA (lateral view) at age 7 years of a child with biallelic loss-of-function mutations in SAMHD1 showing stenoses of terminal internal carotid and basilar arteries with extensive collateral formation (moyamoya).

Intracranial calcification (ICC) is a frequent neuroradiological sign observed in many type 1 interferonopathies (Figure 2A). While ICC is a feature of multiple diseases (37), its presence, and pattern, often serves as an important diagnostic prompt towards the assessment of type 1 interferon signalling (38). White matter disease is also commonly seen in type 1 interferonopathies. Although a pattern of early frontal and temporal lobe swelling giving way to severe frontal and temporal lobe atrophy is essentially pathognomic for classical AGS (particularly related to mutations in *TREX1* (39)), non-specific white matter disease is much more common (Figure 2B), with, remarkably, AGS-associated genotypes observed to account for ∼10% of 664 genetically confirmed cases of childhood leukodystrophy (40). Importantly, neither calcification nor white matter disease are invariably present in the type 1 interferonopathies. Indeed, brain imaging can be completely normal, so that the clinical history may be the only clue to the diagnosis in the absence of agnostic gene sequencing.

Although mutations in any of *TREX1, RNASEH2A/B/C, SAMHD1, ADAR1, IFIH1, LSM11* or *RNU7-1* have been described to cause classical AGS (41, 42), certain mutant genotypes can also be associated with distinct features. Particularly, bilateral striatal necrosis (BSN) is apparently exclusively seen in the context of ADAR1 (adenosine deaminase RNA specific) mutations (43, 44), presenting as a subacute onset (days/weeks) of severe dystonia at a few months of age or in later childhood (Figure 2C, D). Neurological decompensation is often preceded (indeed, possibly triggered) by a noted (but not major) infectious episode (e.g. chicken pox, upper respiratory tract infection, diarrhoeal illness). The recent discovery that ADAR1 inhibits the spontaneous activation of the left-handed Z-nucleic acid sensor ZBP1 (Z-DNA-binding protein 1), which in turn elicits caspase-8-dependent apoptosis and MLKL (mixed lineage kinase domain like pseudokinase)-mediated necroptosis of ADAR1-deficient cells (45–47), might be relevant to the clear destructive aspect of the BSN observed in affected patients. The reason for the selective involvement of the basal ganglia, more specifically the caudate and putamen with sparing of the thalami (48), is unknown. A second genotype-specific presentation is of occlusive cerebrovascular disease (stenosis, aneurysms, moyamoya) as a feature of SAMHD1 (SAM and HD domain containing deoxynucleoside triphosphate triphosphohydrolase)-related disease, sometimes in the absence of any other stigmata (49) (Figure 2E). The risk of intracerebral haemorrhage in this context is real, albeit unquantified, with certain patients undergoing surgical intervention pre-symptomatically.

### Testing

While routine diagnostic testing for enhanced type 1 interferon signalling remains unavailable in most centres (Panel 3), the assessment of thousands of samples in hundreds of patients demonstrates that the measurement of the expression of interferon stimulated genes in patient whole blood represents a reliable assay for the identification of a major proportion of the type I interferonopathies as currently defined (41, 43, 50–54). Digital enzyme-linked immunosorbent assay technology has also allowed for the direct measurement of IFNα protein in blood and CSF (55). These tests have been demonstrated to be of high utility in directing genetic testing, the interpretation of DNA sequence variants and the identification of novel type 1 interferonopathies, including where a link to type 1 interferon signalling had not been previously recognized (e.g. (31)). It is to be hoped that such testing will become more widely available in a clinical diagnostic setting (56). Because dystonia is a common feature of early onset AGS, neurotransmitters are sometimes measured. However, rather than revealing a primary neurotransmitter disorder, an elevation of CSF neopterin can reflect neuroinflammation (57), serving as an important clue to the diagnosis of, and possibly a useful treatment biomarker (58), in certain type 1 interferonopathies. Other biomarkers might include indicators of end organ damage not directly related to the underlying pathogenesis (e.g. neurofilament light and glial fibrillary acidic protein in the case of the CNS).

### Treatments

Before the definition of the underlying molecular defects, type 1 interferonopathies had been largely refractory to a range of immunomodulatory agents, including IL-1β, IL-6, and TNF-α blockers. In contrast, the use of JAK inhibitors, premised on the blockade of type 1 interferon signalling by inhibiting JAK1 (a component of the type I interferon receptor and indispensable for its activity), has demonstrated clear clinical efficacy, particularly at the level of the skin and systemic disease features - indicating that these drugs address a relevant biological pathway (59, 60). Unfortunately, the effects on the neurological disease seen in AGS have been less encouraging, with two factors likely explaining this finding: the late stage in the disease process at which treatment is initiated in most patients (although, even where early/earlier diagnosis has been achieved, onset (61) and/or progression (62) of CNS involvement has been observed while on treatment); and inadequate CNS drug penetration. The concentration of ruxolitinib (61) and baricitinib (62) in the CSF has been consistently found to be only 10% of that in blood. As such, the future use of drugs showing better drug access to the CNS, including the use of intrathecal administration (63) will be important to explore. Anifrolumab, a monoclonal antibody against the type 1 interferon receptor (IFNAR), might be more effective in blocking type I IFN signalling and is now being assessed in clinical practice (64).

### Clinical non-penetrance

Variability in clinical expression is well a recognized phenomenon in AGS (65). Moreover, clinical non-penetrance is an established feature of certain AGS genotypes (in particular, a study of patients with autosomal dominant MDA5 (melanoma differentiation associated protein 5)-associated disease identified 13.5% (10 of 74) of all defined mutation carriers, to be clinically asymptomatic – with seven of these individuals aged greater than 50 years (54)). Given that in routine practice the clinically asymptomatic siblings of an affected child are not normally characterized molecularly, the frequency of clinical non-penetrance related to AGS genotypes inherited as an autosomal recessive trait is unknown. A recent study is of note then in describing five clinically asymptomatic adults, four of whom were homozygous for the A177T mutation in RNASEH2B (ribonuclease H2 subunit B) – the single most common genotype responsible for AGS (66). Clearly, these observations have potential implications when considering the assessment of therapeutic efficacy in future clinical trials and the interpretation of proposed neonatal screening (67, 68).

### Why is the CNS a particular target organ in the type I interferonopathies?

Close to half of the currently defined type 1 interferonopathies are characterised by neurological involvement. Although elevated levels of IFN-α protein are present in the CSF and serum of people with a number of type 1 interferonopathies, the primary site of IFN-α production in AGS (a neurologically focused phenotype) is the CNS and the primary site in STING-associated vasculopathy of infancy (SAVI) (where neurological disease is rare, but asymptomatic ICC has been described (69)) is outside of the CNS (70). Exactly which cells are responsible for the production of type 1 interferons in these disorders remains unclear, with different studies variably indicating a role for astrocytes (71, 72), endothelial cells (73) and microglia (74).

The sequelae of brain exposure to type I interferons and interferon stimulated gene encoded proteins might depend on the neurodevelopmental stage at which such exposure occurs, with the neonatal brain being particularly vulnerable. This point might be relevant to the low frequency of neurological side-effects seen in adults with multiple sclerosis treated with IFN-β (75). More generally, why the brain might be especially susceptible to dysfunction of proteins involved in the homeostasis of type 1 interferon signalling is unclear. Unfortunately, the majority of type 1 interferonopathy mouse models fail to recapitulate the neurological phenotype of the equivalent human disease state. An important exception to this is a murine model of infantile-onset RNaseT2 deficient leukoencephalopathy, a disorder characterised by severe psychomotor impairment, cystic brain lesions, multifocal white matter alterations and cerebral atrophy (neuroradiologically, a remarkable mimic of in utero cytomegalovirus brain infection) (76). Rnaset2 (ribonuclease T2) knockout mice demonstrate upregulation of interferon stimulated genes and IFNAR1-dependent neuroinflammation, with CD8+ effector memory T cell and inflammatory monocyte infiltration into the grey and white matter, and RNA sequencing of single nuclei consistent with homeostatic dysfunction of glial cells and neurons.

While sensing of self-derived nucleic acid can be detrimental, tonic signalling can provide an intrinsic level of basal type 1 interferon signalling. Because of the absence of an endogenous adaptive arm of immunity in the CNS, constitutive innate immune signalling might be particularly important for brain health in post-mitotic neuronal cells with limited capacity for regeneration (77, 78), and also involve other, currently poorly understood, antiviral defence mechanisms (79–81). Such ‘priming’ of neurons might also make them more susceptible to triggers of inflammation – for example, due to mutations in ADAR1, for which Dorrity and colleagues identified the production of RNAs with long 3’UTRs (three prime untranslated regions) as giving rise to exceptionally high levels of immunostimulatory dsRNA structures in neurons (82). In neurons deficient in ADAR1, these dsRNA species triggered MDA5-mediated toxic inflammation and neuronal death. That is to say, inherently high levels of dsRNA in neurons could provide a molecular basis for why inflammation is most prominent in the AGS brain. Of note, these data do not preclude the possibility that glial cells (astrocytes, oligodendrocytes, microglia) could also serve as sources of immunostimulatory dsRNAs.

### Type I IFNs and neurodegeneration

It is now accepted that the brain is not an immune-privileged organ, with the pathological consequences of viral brain infection at least partially explained by the host inflammatory response (83–85). Related to this point, there is growing scientific interest in the possible contribution of enhanced type I interferon signalling to the pathology seen in common forms of neurodegeneration, including Alzheimer’s disease (86, 87), amyotrophic lateral sclerosis (88) and age-related neurodegeneration (89). It is also appropriate to mention here recent data linking triplication of IFNAR1/2 to a type I interferonopathy state in trisomy 21 (90–93). Notably, the high levels of dsRNA in neurons described above (82) might serve as the molecular trigger for chronic PKR (protein kinase R) activation and type 1 interferon induction observed in studies of neurodegenerative disorders including Alzheimer’s disease and amyotrophic lateral sclerosis (94–96). Further, as alluded to earlier, the potential for mitochondrial-derived DNA to drive type 1 interferon mediated neuroinflammation and neurodegeneration has been highlighted by several recent high-profile papers investigating the pathogenesis of Parkinson’s disease (97), Huntington’s disease (98) and amyotrophic lateral sclerosis (99). Type 1 IFN signalling increases pathogen recognition receptor (PRR) expression i.e. PRRs are themselves interferon stimulated genes. Thus, regardless of the initial trigger (e.g., viral infection, self-DNA, self-RNA) or primary source (peripheral, CNS), once the brain is exposed to type I IFN, the risk exists for the induction of a damaging positive feedback loop and chronic inflammation.

## Conclusions and future directions

The discovery of human monogenic disorders underlying either defective or enhanced type 1 interferon activity has delineated the impact of type 1 interferons in natura. Insufficient type 1 interferon predisposes to life-threatening viral disease, with a central role in defence against cerebral infection. In contrast, excessive type 1 interferon appears to underlie a number of autoinflammatory and/or autoimmune phenotypes, the type 1 interferonopathies – many of which affect the CNS. Given the gravity of the biological struggle against external pathogens, and the potential for the misinterpretation of endogenous nucleic acids as non-self, it is unsurprising that type 1 interferon-mediated inflammation has also been implicated in more common forms of neurodegeneration. In highlighting the role of type 1 interferons in brain cellular health, these insights indicate the possibility of ‘anti-interferon’ treatment and, by implication, the importance of identifying a disorder as type I interferon-related.

### Search strategy and selection criteria

References were identified by searches of PubMed (with no date or language restriction) for the terms ‘Aicardi-Goutières syndrome’, ‘interferonopathy’, and ‘interferon + neurodegeneration’. The final reference list was generated on the basis of relevance to the topic, with particular emphasis placed on papers published in the last five years, and a reference limit of 100.

## Panel 1. Stereotyped neurological phenotypes observed in type I interferonopathies

### Classical Aicardi-Goutières syndrome (AGS)

Prenatal or infantile onset, sometimes presenting as a remarkable mimic of trans-placentally acquired infection (pseudo-TORCH syndrome) with microcephaly, irritability, feeding difficulties, abnormal movements and epileptic seizures, as well as haematological disturbance such as thrombocytopenia, anaemia and liver dysfunction, associated with cerebral white matter disease (leukodystrophy), calcification and atrophy on neuroimaging.

### Later onset syndromic spastic-dystonia

Disease presenting beyond the first year of life after apparently normal development to that time, with either the subacute onset of profound neurological regression otherwise similar to classical AGS, or with a more slowly progressive spastic-dystonia variably associated with non-specific white matter changes, intracranial calcification, cerebral atrophy or even normal neuroimaging.

### Bilateral striatal necrosis

Subacute (days/weeks) onset of severe dystonia in the context of bilateral striatal necrosis (symmetrical signal change in the caudate and putamen – but not thalami - associated with swelling and later shrinkage), manifesting at a few months of age or in later childhood, often proceeded (possibly triggered) by a notable - but not major - infectious episode, almost exclusively due to ADAR1 mutations.

### Non-syndromic spastic paraparesis

Slowly progressive spastic paraparesis confined to the lower limbs due to mutations in ADAR1, IFIH1 and RNASEH2B, in the absence of any intellectual deficit, and frequently with completely normal spinal and cranial imaging (or minimal/non-specific high signal T2 deep white matter change +/- cerebral calcification.

### Intracerebral large vessel disease

Moyamoya and aneurysms with an associated (as yet undefined) risk of intracerebral haemorrhage and infarcts, a particular feature of SAMHD1-related disease. Some affected individuals have completely normal psychomotor development (perhaps only demonstrating chilblain-like lesions of the skin), before presenting with a cerebrovascular accident.

## Panel 2. Example clinical histories recorded in patients demonstrating neurological involvement in the context of a type I interferonopathy

### Progressive dystonia

Male beginning to experience problems with holding multiple keys down simultaneously on a computer keyboard when a teenager, thought to represent a repetitive strain injury. At university in his early twenties his symptoms worsened, and he was subsequently diagnosed with an upper limb dystonia. By age 32 years he had developed marked forward flexion of the neck (chin on chest), which showed some improvement following botulinum toxin injection. Cerebral CT revealed bilateral basal ganglia calcification. His disease has been slowly progressive, so that at age 42 years he now has spastic-dystonic involvement of the lower limbs with normal intelligence. He is homozygous for the recurrent p.(Arg177Thr) (c.529G>A) mutation in RNASEH2B (the single most common genotype cause of classical AGS).

### Progressive non-syndromic lower limb spastic paraparesis

Male, who acquired all early milestones appropriately – sitting at age 6 months and walking independently at under 1 year of age. At age 2 years he was noted to be toe-walking and falling more than previously. Between 4 and 15 years of age he underwent multiple tendon lengthening operations. His disorder has been slowly progressive, so that while in his teens he was able to play as goalkeeper for a local football team, by early adulthood he could walk only with the aid of sticks. In his late thirties he demonstrates significant lower limb spasticity, with no involvement of the upper limbs, is cognitively fully intact, and has experienced no other health problems. Brain and spinal imaging at age 29 years was unremarkable. He is heterozygous for a de novo p.Gly495Arg (c.1483G>A) mutation in MDA5 (encoded by *IFIH1*).

### Syndromic spastic paresis

Female, noted to have absent eyelashes and eyebrows, myopia, and early-onset alacrimia in the first year of life. She subsequently developed antibody-positive Hashimoto’s thyroiditis, vitiligo and growth hormone deficiency. Mild lower limb spasticity was noted in infancy, prompting cerebral MRI at age 6 years and a CT one year later, both of which were normal. She attended university with no intellectual deficits into adulthood, but demonstrates a spastic diplegic gait which appears stable. Beginning at 21 years of age she developed sclerodermatous involvement of the hands, face, and ventral surface of the forearms, which progressed rapidly over a few months. Skin biopsy showed classical features of systemic sclerosis. She has a de novo (p.(Gly355Asp) (a c.1064G>A) mutation in ATAD3A.

### Acute bilateral striatal necrosis

Female, demonstrating completely normal development until age 16 months when she experienced an acute loss of all skills over a two-week period. Cranial MRI showed symmetrical signal change in the caudate and putamen, associated with swelling and later shrinkage. By age 2 years she was anarthric, required gastrostomy feeding and had severe spastic dystonic involvement of all 4 limbs. She was found to be compound heterozygous for a recurrent p.(Pro193Ala) (c.577C>G) missense substitution and a p.(Asn857Alafs*17) frameshift mutation in ADAR1.

### Intracerebral occlusive vasculopathy with moyamoya

Female, demonstrating motor delay and four limb spasticity by age 9 months. Cerebral CT showed bilateral basal ganglia calcification. Her head size was normal and remained so subsequently. From childhood she experienced chilblains and Raynaud-type phenomenon, involving the tips of the fingers and toes on exposure to cold with occasional skin breakdown. At age 6 years she had good language, vision and hearing, although she attended a special school and was considered to have moderate learning difficulties. Her mobility was limited by quadriparesis, necessitating the use of a wheelchair. Cerebral MRI at age 7 years revealed severe bilateral stenosis of the supra-clinoid internal carotid and the middle and posterior cerebral arteries, with profuse collaterals around the basal ganglia and thalami, and lacunar infarcts in the right centrum semiovale and left posterior parietal and occipital lobes. These findings were interpreted as an occlusive vasculopathy with secondary moyamoya collaterals. She was started on aspirin and referred for consideration of surgical revascularization. However, before surgery, she suffered a fatal intraventricular haemorrhage. She was homozygous for a p.(Ile201Asn) (c.602T>A) mutation in SAMHD1.

## Panel 3. Interferon signalling assays

### Interferon signature

Assessment of the expression of a panel of representative interferon stimulated genes (ISGs) (initially selected on the basis of microarray and RNA-Seq data). The PAXgene system, with samples travelling stably at room temperature for at least 72 hours, is highly practicable. ISG expression is not specific to any interferon subtype. ISGs cannot normally be assessed in CSF because of low cell numbers.

### Single molecule array

Measurement of interferon alpha protein by digital ELISA in blood and CSF. Costs of the platform/reagents, antibody specificity, and the need to transfer material on dry ice, are important considerations.

*Comments*:

- Depending on the clinical context, serial testing may be necessary to minimise the risk of misinterpretation of a ‘false positive’ result (e.g. due to infection)
- The ability to record a disease signal in blood has proven highly useful, even if this is not necessarily the relevant tissue in terms of underlying pathology
- Although reliable in many genotypes (for example, the interferon signature is positive in close to 100% of patients with *TREX1* mutations), there are exceptions; most particularly, around 20% of patients with mutations in *RNASEH2B* do not show ISG upregulation when tested after age 4 years (41)
- An interferon signature is not disease-specific, being common to a number of apparently distinct phenotypes such as Aicardi-Goutières syndrome, lupus and dermatomyositis
- There is a poor correlation between the level of the expression of interferon stimulated genes and clinical status, most starkly illustrated by individuals with a completely normal phenotype **[**demonstrating, apparently lifelong, upregulation of interferon signalling (e.g. (54, 66))
- While an excellent disease biomarker, our experience is that the interferon score has not behaved as a (highly) reactive biomarker relating to JAK1 inhibition in patients with AGS and *STING1* mutations, at least at the doses used. In contrast, we have seen normalisation of ISG expression in the blood of a number of patients treated with the anti-type I IFN receptor (IFNAR) antibody anifrolumab

## Figures and Tables

**Table 1:** Type 1 interferonopathies demonstrating involvement of the CNS

Protein/RNA (gene)	Function	Proposed link to type I interferon signalling	Mutation effect	Major neurological [and non-neurological] phenotypes (OMIM)	Age at onset, neuroradiological and other clinical features¶
TREX1	Deoxyribonuclease	Cytosolic DNA	Loss of function (autosomal recessive or autosomal dominant-negative)	Aicardi-Goutières syndrome (225750) [CL (610448), systemic lupus erythematosus (152700)]*	Usually first year of life; white matter disease (high T2 signal), intracranial calcification, cerebral atrophy; overall most severe Aicardi-Goutières syndrome genotype
RNASEH2A	Ribonuclease	Cytosolic RNA:DNA hybrids; micronuclear DNA	Loss of function (autosomal recessive)	Aicardi-Goutières syndrome (610333)	Usually first year of life; white matter disease (high T2 signal), intracranial calcification, cerebral atrophy
RNASEH2B	Ribonuclease	Cytosolic RNA:DNA hybrids; micronuclear DNA	Loss of function (autosomal recessive)	Aicardi-Goutières syndrome, spastic paraparesis (610181)	Usually first two years of life; white matter disease (high T2 signal), intracranial calcification, cerebral atrophy; neuroimaging can be non-specific, and even normal; overall least severe Aicardi-Goutières syndrome genotype with some cases presenting as apparently isolated spastic paraparesis
RNASEH2C	Ribonuclease	Cytosolic RNA:DNA hybrids; micronuclear DNA	Loss of function (autosomal recessive)	Aicardi-Goutières syndrome (610329)	Usually first year of life; white matter disease (high T2 signal), intracranial calcification, cerebral atrophy
SAMHD1	Control of dNTP pool / ssRNA 3’ exonuclease	Cytosolic DNA / RNA	Loss of function (autosomal recessive)	Aicardi-Goutières syndrome, cerebrovascular disease (612952) [CL 614415)]	Usually first year of life; white matter disease (high T2 signal), intracranial calcification, cerebral atrophy; particular risk of cerebrovascular disease (moyamoya, aneurysms) and cerebral haemorrhage
ADAR1	RNA editor	Cytosolic dsRNA	Loss of function (autosomal recessive or autosomal dominant-negative)	Aicardi-Goutières syndrome, bilateral striatal necrosis, spastic paraparesis (615010) [dyschromatosis symmetrica hereditaria (127400)]	Usually first year of life; white matter disease (high T2 signal), intracranial calcification, cerebral atrophy; particular association with bilateral striatal necrosis
MDA5 (*IFIH1*)	dsRNA sensor	Cytosolic dsRNA	Gain of function (autosomal dominant)	Aicardi-Goutières syndrome, spastic paraparesis, neuromyelitis optica (615846) [Singleton Merten syndrome (182250)]	Usually first year of life; white matter disease (high T2 signal), intracranial calcification, cerebral atrophy; spastic paraparesis in the absence any other neurological features and normal neuroimaging a particular feature
LSM11	Replication dependent pre-mRNA processing	Histone stoichiometry	Loss of function (autosomal recessive)	Aicardi-Goutières syndrome (619486)	Usually first year of life; white matter disease (high T2 signal), intracranial calcification, cerebral atrophy; particular risk of hypertension and progressive liver and kidney dysfunction
U7 (*RNU7-1*)^	Replication dependent pre-mRNA processing	Histone stoichiometry	Loss of function (autosomal recessive)	Aicardi-Goutières syndrome (619487)	Usually first year of life; white matter disease (high T2 signal), intracranial calcification, cerebral atrophy; particular risk of hypertension and progressive liver and kidney dysfunction
ATM	dsDNA break repair	Cytosolic DNA	Loss of function (autosomal recessive)	Ataxia telangiectasia (208900)	Infantile onset; ataxia with telangiectasia and immunodeficiency
PNPT1	Polynucleotide phosphorylase	Cytosolic mitochondrial RNA	Loss of function (autosomal recessive)	Aicardi-Goutières syndrome-like, bilateral striatal necrosis (610316)	Usually first year of life; Aicardi-Goutières syndrome-like (white matter disease (high T2 signal), intracranial calcification, cerebral atrophy) and some cases presenting with a Leigh syndrome phenotype
ATAD3A	Mitochondrial membrane protein	Cytosolic mitochondrial DNA	Loss of function (autosomal recessive or autosomal dominant-negative)	Global developmental delay, spastic paraparesis [systemic sclerosis] (617183)	Usually first year of life; neuroimaging most frequently normal even where severe developmental delay +/- spastic-dystonic features are present
ARF1	GTPase	STING trafficking / mitochondrial integrity	Molecular mechanism unclear (autosomal dominant)	Global developmental delay [chilblain lupus] (103180)	Usually first year of life; neuroimaging most frequently normal even where severe developmental delay +/- spastic-dystonic features are present
STAT2	Regulation of interferon stimulated gene transcription	IFNAR2 negative feedback signalling	Loss of function (autosomal recessive)	Aicardi-Goutières syndrome-like (600556)	Usually first year of life; white matter disease (high T2 signal), intracranial calcification, cerebral atrophy
ISG15	Inhibition of type 1 interferon receptor signalling	IFNAR2 negative feedback signalling	Loss of function (autosomal recessive)	Intracranial calcification [Mendelian susceptibility to mycobacterial disease] (616126)	Usually first few years of life, presenting with seizures and intracranial calcification
USP18	Inhibition of type 1 interferon receptor signalling	IFNAR2 negative feedback signalling	Loss of function (autosomal recessive)	Aicardi-Goutières syndrome-like (617397)	Usually first year of life; white matter disease (high T2 signal), intracranial calcification, cerebral atrophy
Trisomy 21^^	Trisomy of chromosome 21	Triplication of IFNAR1/2	Gain of function (aneuploidy)	Down Syndrome, intracranial calcification (190685)	Usually first year of life; intracranial calcification is a recognised feature
Complement deficiency**	Alternative complement pathway	Immune complex clearance	Loss of function (autosomal recessive)	Cerebrovascular disease [systemic lupus erythematosus] (613652)	Usually first few years of life, most commonly as systemic lupus erythematosus sometimes with intracerebral vasculitis
Proteasomal related autoinflammatory syndromes (PRAAS)**	Proteasome	PKR stimulation	Loss of function (autosomal recessive or autosomal dominant-negative or autosomal dominant haploinsufficiency or digenic)	Global developmental delay [autoinflammation] (256040)	Multisystem inflammatory disease most often presenting in early childhood; frank neurological involvement is not common but basal ganglia calcification is seen frequently when looked for
TLR7	Endosomal RNA sensor	Endosomal RNA signalling	Gain of function X-linked	Neuromyelitis optica, intracranial calcification [systemic lupus erythematosus] (300365)	Most cases described to date presented in childhood with lupus-like disease and features of neuroinflammation manifesting as intracranial calcification and white matter disease (high T2 signal)
RNASET2	Lysosomal endoribonuclease	Processing RNA ligands of TLR8	Loss of function (autosomal recessive)	Aicardi-Goutières syndrome-like (612951)	Usually first year of life; neuroradiological mimic of congenital cytomegalovirus infection with patchy white matter disease and calcification
ADA2	Adenosine deamination	Undetermined	Loss of function (autosomal recessive)	Cerebrovasculitis [autoinflammation] (615688)	Multisystem inflammatory disease most often presenting in early childhood; neurological involvement is not universal, but when present manifests as cerebrovasculitis with risk of cerebral haemorrhage
TRAP (*ACP5*)	Lysosomal phosphatase	Undetermined	Loss of function (autosomal recessive)	Intracranial calcification, spastic paraparesis [autoimmune haemolytic anaemia, systemic lupus erythematosus] (607944)	Bone dysplasia evident in early childhood, with spasticity manifesting in some individuals in association with intracranial calcification

^ U7 is a non-protein encoding RNA

^^ Trisomy 21 is an aneuploidy, not a single gene, disorder

* While the disorder retinal vasculopathy with cerebral leukoencephalopathy (RVCL) is also caused by mutations in TREX1, there is not evidence that RVCL is associated with enhanced type I interferon signalling

** Both complement deficiency and PRAAS comprise multiple distinct mutant genotypes not listed individually here (the OMIM number relating to the most common mutant genotype is supplied with each of these entries)

¶ Other than trisomy 21, no reliable epidemiological data are available for these genotypes/diseases, but which are all rare (incidence < 1/100,000 live births)
